# Regulation of miRNA expression in the prefrontal cortex by fecal microbiota transplantation in anxiety-like mice

**DOI:** 10.3389/fpsyt.2024.1323801

**Published:** 2024-02-12

**Authors:** Simin Chen, Mengjia Li, Changqing Tong, Yanan Wang, Jiahui He, Qi Shao, Yan Liu, Ying Wu, Yuehan Song

**Affiliations:** ^1^ College of Traditional Chinese Medicine, Beijing University of Chinese Medicine, Beijing, China; ^2^ College of Life Science, Beijing University of Chinese Medicine, Beijing, China; ^3^ Liuzhou Key laboratory of infection disease and immunology, Research Center of Medical Sciences, Liuzhou People's Hospital affiliated to Guangxi Medical University, Liuzhou, Guangxi, China

**Keywords:** generalized anxiety disorder, fecal microbiota transplantation, gut microbiota, prefrontal cortex, miRNA

## Abstract

**Background:**

The gut-brain axis and gut microbiota have emerged as key players in emotional disorders. Recent studies suggest that alterations in gut microbiota may impact psychiatric symptoms through brain miRNA along the gut-brain axis. However, direct evidence linking gut microbiota to the pathophysiology of generalized anxiety disorder (GAD) via brain miRNA is limited. In this study, we explored the effects of fecal microbiota transplantation (FMT) from GAD donors on gut microbiota and prefrontal cortex miRNA in recipient mice, aiming to understand the relationship between these two factors.

**Methods:**

Anxiety scores and gut microbiota composition were assessed in GAD patients, and their fecal samples were utilized for FMT in C57BL/6J mice. Anxiety-like behavior in mice was evaluated using open field and elevated plus maze tests. High-throughput sequencing of gut microbiota 16S rRNA and prefrontal cortex miRNA was performed.

**Results:**

The fecal microbiota of GAD patients exhibited a distinct microbial structure compared to the healthy group, characterized by a significant decrease in Verrucomicrobia and Akkermansia, and a significant increase in Actinobacteria and Bacteroides. Subsequent FMT from GAD patients to mice induced anxiety-like behavior in recipients. Detailed analysis of gut microbiota composition revealed lower abundances of Verrucomicrobia, Akkermansia, Bifidobacterium, and Butyricimonas, and higher abundances of Deferribacteres, Allobaculum, Bacteroides, and Clostridium in mice that received FMT from GAD patients. MiRNA analysis identified five key miRNAs affecting GAD pathogenesis, including mmu-miR-10a-5p, mmu-miR-1224-5p, mmu-miR-218-5p, mmu-miR-10b-5p, and mmu-miR-488-3p. Notably, mmu-miR-488-3p showed a strong negative correlation with Verrucomicrobia and Akkermansia.

**Conclusion:**

This study demonstrates that anxiety-like behavior induced by human FMT can be transmitted through gut microbiota and is associated with miRNA expression in the prefrontal cortex. It is inferred that the reduction of Akkermansia caused by FMT from GAD patients leads to the upregulation of mmu-miR-488-3p expression, resulting in the downregulation of its downstream target gene Creb1 and interference with its related signaling pathway. These findings highlight the gut microbiota’s crucial role in the GAD pathophysiology.

## 1 Introduction

The gut-brain axis and gut microbiota have emerged as crucial elements in the pathophysiology of neurological and psychiatric diseases. Increasing empirical evidence supports the notion that the bidirectional communication between gut microbiota and the central nervous system is linked to emotional disorders, including anxiety (1), with generalized anxiety disorder (GAD) being the most prevalent anxiety disorder (2). Recent studies have demonstrated that gut microbes impact brain function, including the prefrontal cortex, through neural, immune, and endocrine pathways, leading to the onset of adverse emotions such as anxiety. This influence includes the direct activation of the vagus nerve by neurotransmitters released by gut flora, interaction with gut-derived immune mediators and immune cells causing microglia activation and neuroinflammation, and the production of metabolites by gut microbes that regulate brain function (3, 4). Current evidence suggests that neuroinflammation induced by a high-cholesterol diet can induce anxiety and depression behavior in mice through alterations in gut microbiota, specifically a reduction in Akkermansia (5, 6), while supplementing Akkermansia can significantly alleviate anxiety and depression symptoms in mice subjected to chronic stress (6). Similarly, depleting gut microbiota using antibiotics has demonstrated the ability to ameliorate abnormal emotional behavior (7). Clinical studies have reported reduced gut microbiota richness and diversity in patients with GAD (8), with the enrichment of Bacteroides positively correlated with anxiety severity (9). Accordingly, the gut microbiota has emerged as a potential biomarker for studying the pathogenesis of mental disorders and may offer new treatment targets for anxiety-related diseases.

Chronic stress and anxiety lead to the continuous secretion of excessive neurotransmitters and hormones, such as adrenaline and cortisol, causing damage to the brain, particularly the prefrontal cortex. This damage results in the death of neurons, reducing the size and function of the prefrontal cortex (10). MicroRNAs (miRNAs) in the prefrontal cortex play a regulatory role in the expression of genes associated with generalized anxiety disorder (11), influencing the balance and signaling of neurotransmitters like gamma-aminobutyric acid or glutamate, ultimately affecting mood and behavior (12). Notably, miR-323a-3p has been shown to play a significant role in the regulation of severe depression (13), while miR-144-5p levels have been associated with depression, anxiety, or stress-related phenotypes (14). Furthermore, plasma levels of miRNA-132 and miRNA-134 can serve as biomarkers for the detection of obsessive-compulsive disorder (15), highlighting the potential for miRNAs to serve as regulatory molecules involved in the prediction and diagnosis of mental disorders.

Recent studies highlight the intricate interplay between gut microbiota and miRNAs in the brain in the occurrence and development of anxiety behavior. The gut microbiota is suggested to regulate the expression of specific miRNAs, thereby influencing brain function and behavior. For instance, mouse gut dysbiosis result in abnormal anxiety-like behavior and alterations in miRNA expression profiles in the prefrontal cortex and amygdala, which normalize upon reintroduction of gut microbiota (16). To investigate the pathogenesis of GAD, we posited that gut flora sourced from GAD patients induces anxiety-like behaviors by modulating miRNA expression in the prefrontal cortex of mice through the gut-brain axis. In this experimental approach, we initially established pseudo-germ-free model mice through a standardized antibiotic regimen, achieving a near-sterile bacterial load in the gut. These mice could reflect the biological functions and mechanisms of the newly transplanted microbiota (17), displaying analogous changes in signaling pathways and organ morphology to those observed in sterile mice (18). Subsequently, fecal samples obtained from both GAD patients and healthy individuals were processed into fecal bacterial mixtures and transplanted onto pseudo-germ-free mice, establishing an experimental animal model with a humanized flora. This model was designed to explore the gut microbiota characteristics associated with GAD. The prefrontal cortex, a central brain region implicated in emotional cognition related to anxiety, was chosen for miRNA sequencing to identify critical miRNAs associated with the microbiota. This targeted approach aimed to unveil novel biomarkers of GAD, offering a pivotal research strategy for elucidating the core pathological mechanisms underlying this disorder.

## 2 Materials and methods

### 2.1 Participant recruitment

The protocol outlined in this manuscript received approval from the Medical and Experimental Animal Ethics Committee of Beijing University of Chinese Medicine. Five individuals diagnosed with GAD, aged between 18 and 60, were recruited from Beijing Anding Hospital, affiliated with Capital Medical University. Five age-matched controls with no history of major diseases were recruited from the local community. Inclusion criteria for GAD patients comprised (1) meeting diagnostic criteria for GAD according to the fifth edition of the Diagnostic and Statistical Manual of Mental Disorders (DSM-5), (2) obtaining a Hamilton Anxiety Scale score equal to or greater than 14 points, and (3) experiencing a first episode without prior use of anti-anxiety medications. Exclusion criteria for GAD patients included (1) presence of other mental health conditions or substance abuse, (2) consumption of probiotic fermented foods within less than three days before the study initiation, and intake of antibiotics, probiotics, or prebiotics within less than 14 days before the study initiation, and (3) severe chronic illnesses or infections, with a body mass index (BMI) exceeding 28.0. All participants provided written informed consent.

Fecal samples were obtained from the subjects during their first bowel movement in the morning and were promptly stored in a -80°C freezer. To ensure uniformity, the stool specimens were meticulously mixed, and 20g portions were precisely measured. The samples were then thawed on ice and diluted with 200 mL of pre-cooled sterile saline. The mixtures underwent vigorous shaking for 5 minutes, followed by filtration through a fine mesh, and allowed to settle for an additional 5 minutes. Subsequently, approximately 108mL of the resulting clear supernatant was aspirated and thoroughly mixed to create a fecal bacteria suspension. The feces from healthy individuals and GAD patients were separately mixed to obtain 1 tube each of fecal suspension from healthy individuals and fecal suspension from GAD patients. Subsequently, they were analyzed by 16S rRNA high-throughput sequencing for further analysis.

### 2.2 Scales

The assessment of anxiety levels in individuals with GAD and healthy controls commonly employs the 7-item Generalized Anxiety Disorder Questionnaire (GAD-7), Hamilton Anxiety Scale (HAMA), and Self-Rating Anxiety Scale (SAS). The reliability and validity of the Chinese versions of these three scales have been established (19). The GAD-7, a 7-item questionnaire, utilizes a Likert scale ranging from 0 to 3 to evaluate the frequency of anxiety symptoms. A cutoff score of 10 or above is commonly utilized for screening purposes. HAMA, a 14-item scale, assesses anxiety severity on a scale from 0 to 4, with a score of 14 or above indicative of the presence of anxiety. SAS, a 20-item self-report instrument, employs a Likert scale from 1 to 4 to assess anxiety symptoms based on frequency. Total scores are derived by summing all item scores and multiplying by 1.25 for normalization. Generally, SAS scores are higher in individuals with greater anxiety severity.

### 2.3 Animals

Specific-pathogen-free (SPF) male C57BL/6J mice, aged 6-8 weeks and weighing between 18-22g, were procured from Beijing Vital River Laboratory Animal Technology Co., Ltd (Permission NO.SCXK (jing) 2021-0006). The mice were housed in the animal facility at Beijing University of Chinese Medicine. The food and water provided for mouse feeding underwent sterilization through high-pressure methods. The housing environment was carefully maintained within a temperature range of 22 to 24 degrees Celsius, with a light cycle consisting of 12 hours of light and 12 hours of darkness and a relative humidity maintained at 60% to 70%. Following a period of 7 days for adaptive housing, the mice were subjected to formal experimental procedures. Animal experiments were reviewed and approved by the Laboratory Animal Ethics Committee of Beijing University of Chinese Medicine (Ethical Committee Approval Code: BUCM-4-2021051203-2077).

### 2.4 FMT regime

The mice were randomly allocated into three groups (n = 7 for each group): control (no antibiotic treatment, no FMT), FMT health (antibiotic treatment followed by FMT from healthy donors), and FMT GAD (antibiotic treatment followed by FMT from GAD patients). The formal experiment extended over a period of 29 days. Antibiotics, including metronidazole (1 mg/mL), ampicillin (1 mg/mL), neomycin sulfate (1 mg/mL), and vancomycin (0.5 mg/mL), were administered orally to each mouse at a dosage of 0.1 ml/10 g once daily at 9:00 am for a consecutive 14 days. Subsequently, on day 15, daily FMT was conducted at 9:00 am by administering fecal suspensions through oral gavage to each mouse at a dosage of 0.1 ml/10 g every three days, totaling five administrations over 15 days.

### 2.5 16S rRNA gene sequence analysis

The study employed a kit-based approach by Microeco Tech Co., Ltd. (Shenzhen, China) for DNA extraction and 16S rRNA sequencing from fecal samples. Total DNA extraction was conducted, and its concentration, purity, and quality were assessed using NanoDrop2000 and 2% agarose gel electrophoresis, respectively. The V3-V4 variable region was amplified through PCR, and the size of the amplification target band was determined using 1% agarose gel electrophoresis. PCR automation purification was performed using the magnetic bead method. A specific volume of PCR product from each sample was used to create a machine library, and library construction was completed after screening and PCR enrichment. Finally, Illumina’s Miseq PE300 platform was employed for sequencing.

As the sequencing object involves single-sample data, conventional analyses such as α diversity analysis, β diversity analysis, and Linear discriminant analysis effect size(LEfSe)analysis were not applicable to human fecal samples. Instead, an analysis method similar to miRNA high-throughput sequencing was adopted. This method utilized the number of intestinal flora sequencing entries to calculate the ratio of GAD patients to healthy individuals. Intestinal flora with a sequencing entry count < 5 and |Log2 FoldChange| > 1 were considered to exhibit significant differences between healthy individuals and GAD patients. In contrast, for mouse fecal samples, conventional 16S rRNA gene sequence analysis was employed.

### 2.6 Open field test

Before the experiment, mice underwent a 30-minute habituation period in a behavioral laboratory. The open field box used had dimensions of 50 × 50 × 50 cm³, and a camera was positioned directly above it. During the experiment, mice were placed in the center of the open field box, and their movements were recorded for 5 minutes. Following the recording session, the mice were removed, and the inner walls and floor of the open field box were meticulously cleaned using 75% alcohol. The primary parameters assessed included the total distance traveled, mean speed, duration spent in the central square, and the number of times traversing the square. Ethovision XT 15 software (Noldus, Netherlands) was employed to analyze the footage for specific parameters, including total distance moved, mean velocity, and time spent in the center.

### 2.7 Elevated plus maze test

The elevated plus maze test comprised two closed arms and two open arms, each measuring 65 × 5 cm, with a central open area of 5 × 5 cm. The entire maze was elevated 50 cm above the ground. At the onset of the experiment, mice were positioned in the central open area with their heads facing the open arm, and their behavior was recorded for 5 minutes. The criterion for a recorded entry was met when both forepaws of the mouse entered an arm. Following each mouse’s recording, the maze apparatus was cleaned with 75% alcohol. The observed indicators included the percentage of entries into the open arms and time spent in the open arms. EthoVision XT 15 software (Noldus, Netherlands) was utilized for data analysis. The percentage of entries into the open arms was calculated as follows: number of entries into the open arms/(number of entries into the open arms + number of entries into the closed arms). Similarly, the percentage of time spent in the open arms was calculated as follows: time spent in the open arms/(time spent in the open arms + time spent in the closed arms).

### 2.8 Prefrontal cortex miRNA sequencing and analysis

After completion of the behavioral tests, the prefrontal cortex tissue from the mice was promptly placed on ice and stored at -80°C for subsequent miRNA high-throughput sequencing analysis. Utilizing the NEBNext^®^ Multiplex Small RNA Library Prep Set for Illumina^®^ (NEB), total RNA was isolated from the prefrontal cortex (randomly selecting 5 samples from each group) to prepare a small RNA library. Subsequently, PCR amplification was conducted using LongAmp Taq 2X Master Mix, Illumina’s SR primers, and index (X) primers. The library’s quality was assessed using the Agilent Bioanalyzer 2100 system, and the constructed library was sequenced on the Illumina NovaSeq 6000 platform. Raw sequences of small RNA and transcriptome libraries were compared using MiRBase V20 (http://www.mirbase.org).

The miRNA sequencing results were analyzed using the R package DESeq2 (version 3.5.2) with count-based RNA-seq data as input. DESeq2, employing a negative binomial generalized linear model, was used to detect differential expression, incorporating data-driven prior distributions for dispersion and log-fold changes (20). Differentially expressed miRNA and gene transcripts identified by the DESeq2 method, adjusted for Benjamin-Hochberg false discovery rate with P < 0.05, were considered statistically significant. The miRwalk3.0 software (http://mirwalk.umm.uni-heidelberg.de/) was then employed to predict target genes associated with differentially expressed miRNAs, utilizing the miRwalk and Targetscan databases (21). Subsequently, Cytoscape 3.8.0 software was used to construct a miRNA-gene network, retaining miRNAs with a Degree >10 and their corresponding predicted target genes (21). Additionally, Metascape (https://metascape.org/gp/index.html#/main/step1) was utilized for gene ontology (GO) analysis and exploration of the Kyoto Encyclopedia of Genes and Genomes (KEGG) database to characterize functionally enriched categories and potential signaling pathways involved.

### 2.9 Statistical analysis

The results were expressed as means ± standard deviation. Statistical analysis was performed using SPSS 20.0 software. The significance of participants’ anxiety levels was assessed using the two independent samples t-test. One-way ANOVA was applied to evaluate the statistical significance of behavioral data. For the analysis of the relative abundance and diversity index of bacteria, Kruskal-Wallis and Wilcoxon tests were employed. The correlation between gut microbiota and miRNA was examined using Spearman correlation analysis conducted with Wekemo Bioinclude software (https://www.bioincloud.tech/). Graphical representation of statistical data was generated using GraphPad Prism 8.4.

## 3 Results

### 3.1 Gut microbiota profile is altered in patients with GAD

To characterize the gut microbiota of patients with GAD, we recruited 10 study participants, including 5 GAD patients and 5 healthy individuals. The anthropometric measurements were comparable between the two groups of study participants. The GAD group showed a significant increase in anxiety levels compared to the health group (**Table 1**).

**Table 1:** Anthropometric measurements and clinical characteristics of the study participants.

	Health (n=5)	GAD(n=5)	p
BMI (kg/m2)	20.45 ± 2.72	22.11 ± 1.46	0.264
Heart rate (beats/min)	69.20 ± 10.64	83.00 ± 16.43	0.154
Respiration (beats/min)	17.00 ± 1.00	18.40 ± 2.61	0.295
GAD-7	1.20 ± 1.30	12.00 ± 4.24	0.001
HAMA	2.40 ± 1.14	30.60 ± 5.90	0.000
SAS	29.25 ± 2.09	62.00 ± 8.51	0.001

There was no significant difference in BMI, heart rate, and respiration in the two groups. GAD patients exhibited elevated anxiety levels as indicated by GAD-7, HAMA, and SAS scores. Data are expressed as Mean ± SD; significance testing was assessed by paired t-test.

**Figure 1:**
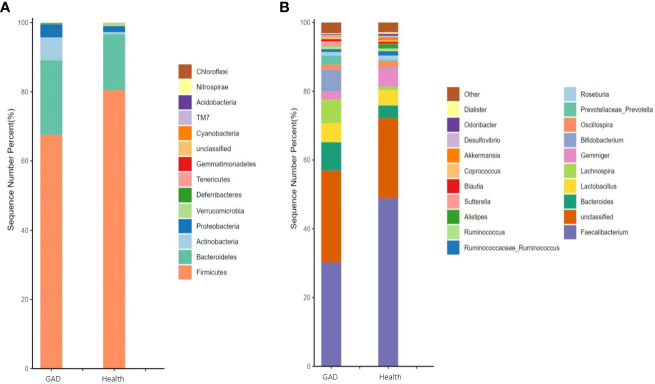
Comparison of the gut microbiota of GAD patients and healthy people. **(A)** Abundance of gut microbiota at the phylum level and different phyla between the groups. **(B)** Abundance of gut microbiota at the genus level and significantly different genera between the groups. (n =1/group).

As described in section 2.4, we employed the number of gut microbiota sequencing entries to perform a structural analysis of the human gut microbiota in patients with GAD compared to healthy individuals. At the phylum level, Actinobacteria, Gemmatimonadetes, and Deferribacteres exhibited a significant upregulation in the GAD group when compared to the healthy group, whereas Verrucomicrobia showed a significant downregulation (**Figure 1A** and **Supplementary Table 1**). On the genus level, Sutterella, Megamonas, Prevotella, Lachnospira, and Bacteroides demonstrated a significant upregulation in the GAD group compared to the healthy group. Conversely, Alistipes, Akkermansia, Butyricimonas, and Parabacteroides were significantly downregulated (**Figure 1B** and **Supplementary Table 2**). These findings suggest notable alterations in the intestinal microbiota characteristics of individuals with Generalized Anxiety Disorder.

### 3.2 Gut microbiota from GAD patients is sufficient to promote anxiety-like behavior in mice

**Figure 2:**
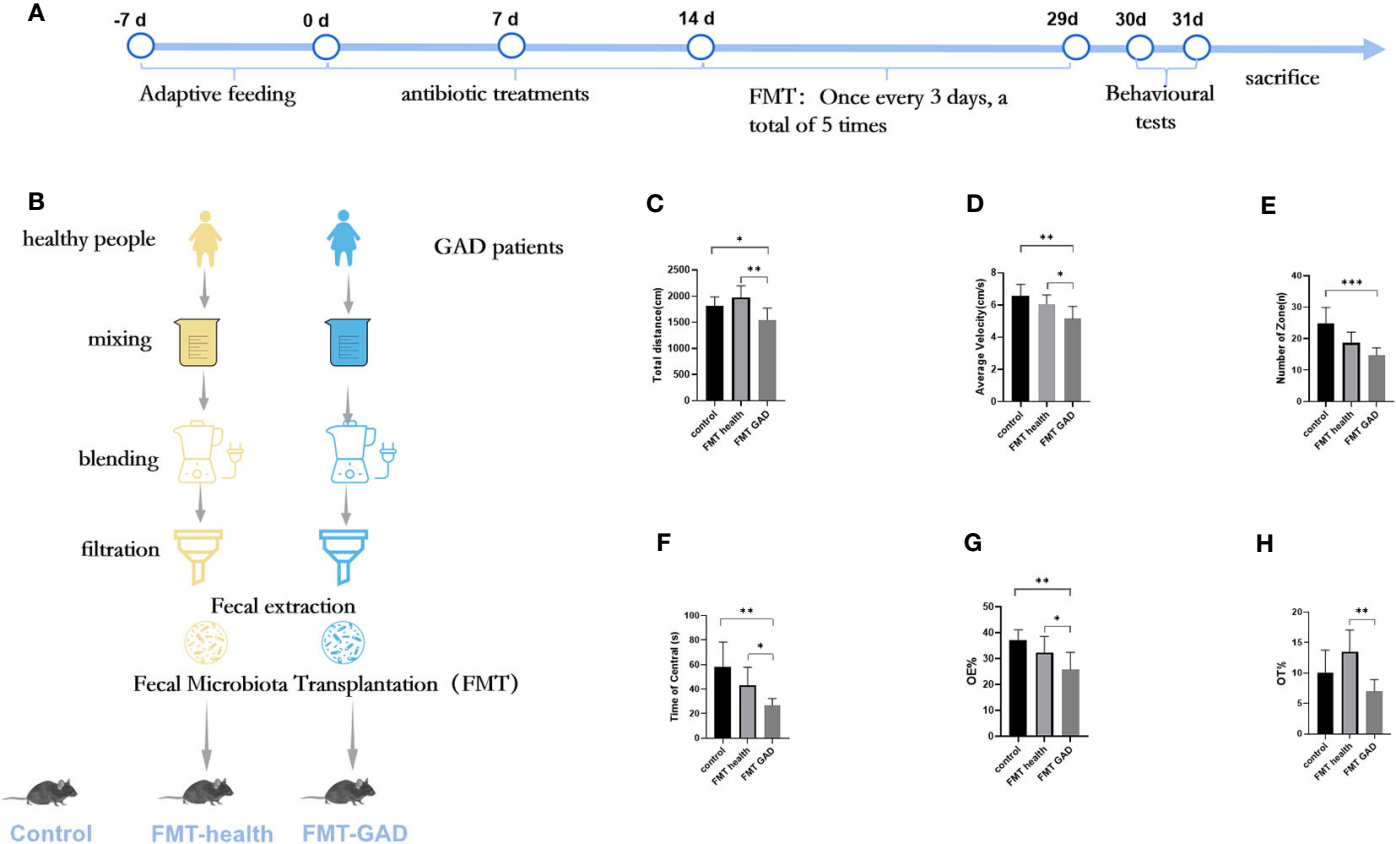
The results of OFT and EPM of mice in each group. **(A, B)**: Experimental design; **(C)**: Total distance in OFT; **(D)**: Average velocity in OFT; **(E)**: Number of zones in OFT; **(F)**: Time spent in the central area in OFT; **(G)**: Percentage of entries into the open arms (OE%) in EPM; **(H)**: Percentage of time spent in the open arms (OT%) in EPM. Compared with the anxiety model group, *P<0.05, **P<0.01, ***P<0.001. Data are presented as mean ± SD (n = 7/group).

To explore whether the transplantation of human gut microbiota could impart anxiety-like behavioral traits from GAD patients to germ-free (GF) mice, we conducted fecal transplantation experiments using samples from both GAD patients and healthy individuals, resulting in the generation of “humanized microbiota” mice, as illustrated in **Figures 2A, B**. Subsequently, on the second day post-FMT, open field tests and elevated plus maze experiments were performed. In the open field experiment, FMT GAD mice exhibited significantly lower total distance moved (P<0.05), average speed (P<0.01), central grid dwell time (P<0.05), and number of grid crossings (P<0.001) compared to both the control and FMT health groups (P<0.01) (**Figures 2C–F**). In the elevated plus maze experiment, FMT GAD mice displayed a significantly lower percentage of entries into open arms (P<0.05) and time spent in open arms (P<0.01) compared to the FMT health group. Additionally, FMT GAD mice had a significantly lower percentage of entries into open arms compared to the control group (P<0.01), and a decreasing trend in the percentage of time spent in open arms was observed (**Figures 2G, H**). These findings collectively suggest that fecal transplants from GAD patients induced anxiety-like changes in the behavior of the mice.

### 3.3 Gut microbiota from GAD patients is sufficient to promote intestinal dysbiosis in mice

**Figure 3:**
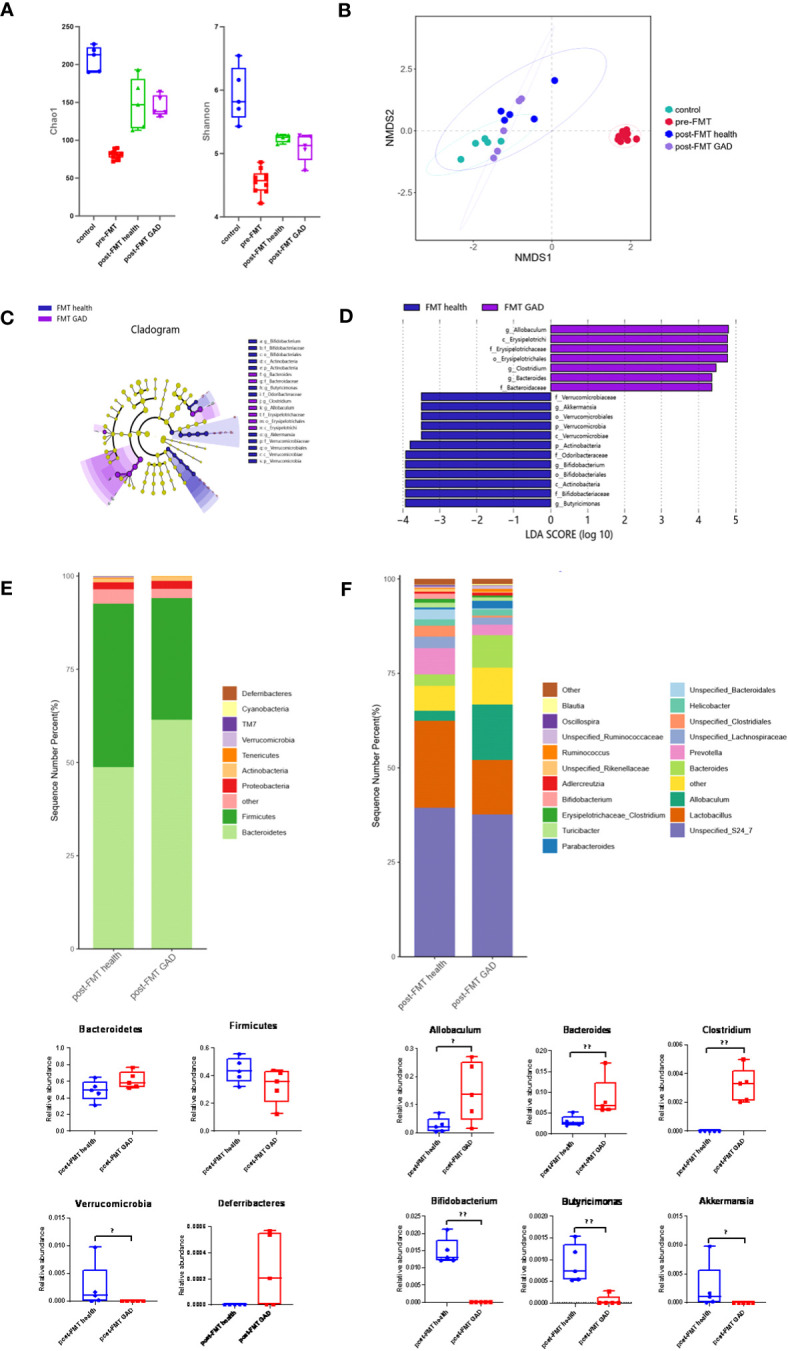
Impact of FMT on gut microbiota composition. **(A)** Measures of alpha diversity among the control group (n=5), pre-FMT group (n=10), and the mice after FMT with healthy (n=5) and GAD (n=5) feces. **(B)** NMDS plot based on Bray Curtis distance. **(C)** Differentially expressed taxa identified by LEfSe analysis between the groups (The threshold of the logarithmic linear discriminant analysis score was >2.0). **(D)** Cladogram from LEfSe analysis, representing the classification level from phyla to genera. **(E)** Abundance of gut microbiota at the phylum level and different phyla between post-FMT health group and post-FMT GAD group (n=5/group). Bacteroidetes (P=0.076>0.05), Firmicutes (P=0.175>0.05), Verrucomicrobia (P=0.019<0.05), Deferribacteres (P=0.054>0.05). **(F)** Abundance of gut microbiota at the genus level and significantly different genera between post-FMT health group and post-FMT GAD group (n=5/group). Allobaculum (P=0.047<0.05), Bacteroides (P=0.009<0.01), Clostridium (P=0.005<0.01), Bifidobacterium (P=0.005<0.01), Butyricimonas (P=0.007<0.01), Akkermansia (P=0.019<0.05). Data are presented as medians.

As previously detailed, control mice received no treatment, while others underwent FMT from either healthy donors or patients with GAD after antibiotic treatment. Fecal samples were obtained from control mice and mice before and after FMT to characterize microbial community features. Alpha diversity analysis revealed that compared to control mice, the Chao1 and Shannon indices of pre-FMT mice (antibiotic-treated mice) were significantly reduced, indicating a notable decrease in the diversity of gut microorganisms due to antibiotic treatment. Following FMT, both post-FMT health and post-FMT GAD mice showed significantly higher Chao1 and Shannon indices, indicating an increase in bacterial load and restoration of microbial diversity to some extent. However, there was no significant difference between these indices in the two groups of mice after FMT (**Figure 3A**). Non-Metric Multidimensional Scaling (NMDS) analysis (Bray–Curtis distance, **Figure 3B**) revealed a clear separation between pre- and post-FMT samples, indicating significant differences in the gut microbial community structure after FMT in both groups (PERMANOVA analysis, P < 0.05). This suggests that mice transplanted with microbiota from normal individuals and anxiety patients harbored significant differences in their gut microbial community structure.

LEfSe analysis identified 19 taxa as potential microbial biomarkers, with 7 from the FMT GAD group and 12 from the FMT health group (**Figures 3C, D**). At the phylum level, Verrucomicrobia and Actinobacteria were the differential bacterial groups in the FMT health group, while not shown in the FMT GAD group. At the genus level, Akkermansia, Bifidobacterium, and Butyricimonas were the differential bacterial groups in the FMT health group, while Allobaculum, Bacteroides, and Clostridium were the differential bacterial groups in the FMT GAD group. Further analysis of fecal microbiota profiles at the phyla and genera levels revealed that Bacteroidetes and Firmicutes were the most abundant phyla, constituting over 90% of all microbial compositions. Although no significant differences were observed between the two groups in these phyla, mice transplanted with GAD microbiota showed an increasing trend in Bacteroidetes and a decreasing trend in Firmicutes compared to mice transplanted with healthy human microbiota. Additionally, Deferribacteres significantly decreased, while Verrucomicrobia showed a significant decrease in the FMT GAD group (**Figure 3E**). At the genus level, six genera exhibited significant differences in abundance between the two groups: Allobaculum, Bacteroides, and Clostridium increased significantly in mice transplanted with GAD patient microbiota, while Bifidobacterium, Butyricimonas, and Akkermansia decreased significantly (**Figure 3F**). These findings suggest that the transplantation of microbiota from anxious patients can induce intestinal dysbiosis.

### 3.4 miRNA high-throughput sequencing results

**Figure 4:**
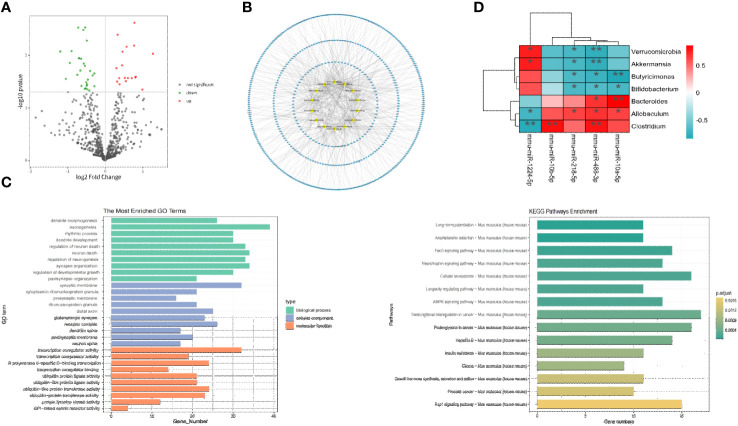
Correlation analysis between key miRNA and key bacterial groups. **(A)** Volcano plot illustrating differential expression of miRNA between two groups (P < 0.05). **(B)** Network plot displaying target genes with a Degree > 10 and their corresponding miRNAs. **(C)** GO and KEGG enrichment analysis to provide insights into the functional significance of the identified miRNA-mRNA interactions. **(D)** Heatmap depicting the correlation analysis between significantly differential bacteria and miRNA. The horizontal axis represents different miRNA, the vertical axis represents different bacteria, and the legend on the right indicates the correlation coefficient. Red indicates a positive correlation, while blue indicates a negative correlation. *P<0.05, **P<0.01.

To identify differentially expressed miRNAs between the two FMT groups, high-throughput sequencing was conducted on prefrontal cortex tissue. The results revealed significant differences in 30 miRNAs (P<0.05). Compared to the FMT health group, the FMT GAD group exhibited 19 upregulated and 11 downregulated miRNAs. The miRNAs with significant expression differences in the two groups are depicted in **Figure 4A**, and details are provided in **Table 2**.

**Table 2:** Differential expression of miRNA between the two groups.

miRNA	p-value	Fold Change	FMT health/FMT GAD
mmu-miR-10b-5p	0.00851991	2.293195532	Up
mmu-miR-539-5p	0.028232906	2.068462126	Up
mmu-miR-10a-5p	0.040375879	1.982540581	Up
mmu-miR-300-5p	0.01402221	1.923686848	Up
mmu-miR-146a-5p	0.008380554	1.862490355	Up
mmu-miR-146b-5p	0.023593799	1.674312816	Up
mmu-miR-148a-3p	0.002982898	1.645267964	Up
mmu-miR-467a-5p	0.011426646	1.62567188	Up
mmu-miR-467b-5p	0.011426646	1.62567188	Up
mmu-miR-335-3p	0.019592116	1.58203147	Up
mmu-miR-451a	0.011681076	1.5605543	Up
mmu-miR-30e-3p	0.038405488	1.553232725	Up
mmu-miR-673-5p	0.027339271	1.519644001	Down
mmu-miR-3470b	0.027236114	1.631896553	Down
mmu-miR-1224-5p	0.006577686	1.704261841	Down
mmu-miR-3102-3p	0.002415015	1.712992118	Down
mmu-miR-690	0.026490287	1.74437906	Down
mmu-miR-744-5p	0.025712589	1.756092883	Down
mmu-miR-3470a	0.04500042	1.985240573	Down
mmu-miR-6538	0.009420542	2.410355205	Down

Only the data with | Foldchange | > 1.5 is shown in the table. For a comprehensive overview, refer to **Table S3** for all contents.

### 3.5 Bioinformatics analysis of target genes results

The prediction of target genes was carried out through the miRWalk 3.0 website. Among the 30 miRNAs with P < 0.05, 22 miRNAs and 1072 target genes were selected through Targetscan and miRWalk databases. As illustrated in **Figure 4B**, miRNAs with a Degree > 10 in the miRNA-target interaction and their corresponding target genes were chosen as key analysis objects. The results of miRNA-Gene Ontology (GO) analysis are presented in **Figure 4C**, revealing that key miRNAs primarily participate in biological processes (BP) such as axonogenesis, neuron death, neuron regulation, dendritic development, and rhythmic processes; in cellular components (CC) such as synapse, glutamatergic synapse, distal synapse, postsynaptic membrane, and dendritic spine; and in molecular functions (MF) such as transcriptional coactivator activity, RNA polymerase II transcription factor activity, protein transferase activity, ubiquitin-like protein ligase activity, etc. According to miRNA-KEGG analysis, key miRNAs are involved in regulating anxiety through pathways like long-term potentiation, AMP-activated protein kinase (AMPK) signaling pathway, cyclic AMP (cAMP) signaling pathway, axon guidance, PI3K-Akt signaling pathway, Dopaminergic synapse, etc.

### 3.6 miRNA-gene network analysis results

14 key miRNAs with relevance scores >10 were selected for focused analysis of the miRNA-target gene relationships. These key miRNAs include mmu-miR-148a-3p, mmu-miR-378c, mmu-miR-1224-5p, mmu-miR-10a-5p, mmu-miR-218-5p, mmu-miR-106b-5p, and mmu-miR-384-5p (**Table 3**).

**Table 3:** miRNA-Gene Degree and corresponding gene.

miRNA	Degree	Gene
mmu-miR-148a-3p	105	Pten, Styx, Scaf11, Usp7, Ago1, Cdk13, Arhgef17, Arpp19, Usp47, Masp1, Mpped1, Sbno1, Fez2, etc.
mmu-miR-378c	69	Nme6, Psd3, Dcaf12, Chtf8, Nup160, Plekha1, Mpp3, Dyrk1a, Gja8, Dlk1, Fkbp5, Htr1b, Igf1, etc.
mmu-miR-1224-5p	68	Creb1, Ptprf, Rtl5, Ush1g, Prickle2, N6amt1, Syt14, Med20, Prpf40a, Dhx35, Prc1, Cbx3, etc.
mmu-miR-10a-5p	53	Flt1, Prrx1, Gnrhr, Wdr26, Slc6a19, E2f3, Ppargc1b, Mapre1, Epha5, Grin2b, Myt1l, Pten, etc.
mmu-miR-218-5p	45	Fam126b, Rps6ka3, Ugt8a, Ssr1, Gpam, Npas2, Pde7a, Camk4, Epha7, Prlr, Sgcd, Ppp1cc, etc.
mmu-miR-10b-5p	45	Med1, Tiam1, Rc3h2, Zmynd11, E2f3, Kctd16, Mapre1, Epha5, Gabrb2, Hoxb3, Elavl3, Irs1, etc.
mmu-miR-384-5p	43	Lcor, Atp6v0d1, Capza1, Kctd16, Capn5, Ell, Fst, Map3k5, Ppp3ca, Strbp, Zfand5, Celf2, etc.
mmu-miR-488-3p	37	Grin2b, Mecom, Rad51d, Fign, Rspry1, Mat2a, Cbx5, Gabrb2, Lhx6, Rab7, Rbpj, Calm1, etc.
mmu-miR-146a-5p	36	Strbp, Meis1, Myo5a, Usp47, Kctd15, Lcor, Foxo3, Kdm2b, Ehf, Itm2b, Syt1, Traf6, etc.
mmu-miR-146b-5p	23	Kctd15, Zfp532, Strbp, Ehf, Gabrb1, Smad4, Angptl2, Ar, Tef, Ube2d2a, Sfpq, Primpol, etc
mmu-miR-137-3p	14	Nab1, Pakap, Ptgfrn, Baz1a, Syncrip, Rnf157, Foxp1, Mosmo, Wbp1l, Sez6l2, Tfap2a, Creb5, Pafah1b2, Epha7
mmu-miR-382-3p	14	Nrp1, Ccnt1, Map3k19, Foxb1, Fgf16, tga1, Atp13a3, Fam168b, Kcnma1, Camk1d, Sepsecs, 1700025G04Rik, Ntrk3, Tfcp2
mmu-miR-744-5p	13	Nrgn, Unc5a, Hes3, Rfx1, Mmp24, Tmem167b, Rab14, Camk2n2, Sh3bgrl3, Vgf, Sbf1, Vps37d, Zfp385a
mmu-miR-504-5p	11	Rnf114, Dcx, Fem1a, Pla2g2f, Crtam, Cutc, Nlrc3, Epb41, Ppargc1b, Ctdspl, Cep170

This table shows only the data with Degree >10. For a more comprehensive assessment, refer to **Table S4** for all contents.

### 3.7 Gene-miRNA-pathway analysis results

In the gene-miRNA analysis, 8 key target genes were identified as regulated by the corresponding miRNAs (**Table S5**). The intersection of these 8 target genes with the genes obtained from the KEGG analysis results was determined, and the resulting intersection genes were enriched in biological pathways related to anxiety in this study. A total of 3 key target genes, regulated by the corresponding miRNAs, were involved in anxiety-related pathways, with Creb1 being the most critical as it was enriched in 4 anxiety-related pathways (**Table 4**). Based on the information from **Tables 3**, **4**, and **S5**, mmu-miR-10a-5p, mmu-miR-1224-5p, mmu-miR-218-5p, mmu-miR-10b-5p, and mmu-miR-488-3p were selected as the focus of subsequent analysis.

**Table 4:** Gene-miRNA Degree, corresponding miRNA, and pathways involved in anxiety.

Target Gene	Degree	FMT health/FMT GAD	Pathways involved in anxiety
Creb1	3	mmu-miR-10a-5p↑、mmu-miR-1224-5p↓、mmu-miR-488-3p↑	AMPK signaling pathway, cAMP signaling pathway, PI3K-Akt signaling pathway, Dopaminergic synapse
Epha5	3	mmu-miR-10a-5p↑、mmu-miR-10b-5p↑、mmu-miR-218-5p↑	Axon guidance
E2f3	3	mmu-miR-10a-5p↑、mmu-miR-10b-5p↑、mmu-miR-384-5p↑	Cellular senescence

### 3.8 Prefrontal cortex miRNAs are strongly associated with gut microbiota

To explore the relationship between gut microbiota and miRNAs, Spearman correlation analysis was conducted between the aforementioned 5 key miRNAs and differential flora at the phylum level. A difference was considered significant when R > 0.6 or R < -0.6 and P < 0.05 (refer to **Figure 4D**). The analysis revealed 13 negative correlation pairs and 8 positive correlation pairs. At the phylum level, mmu-miR-1224-5p was positively correlated with Verrucomicrobia and negatively correlated with mmu-miR-218-5p and mmu-miR-488-3p. At the genus level, mmu-miR-218-5p and mmu-miR-488-3p were negatively correlated with Akkermansia, Butyricimonas, and Bifidobacterium, and positively correlated with Allobaculum, Bacteroides, and Clostridium. Mmu-miR-1224-5p exhibited an opposite relationship with Allobaculum and Clostridium and was positively correlated with Akkermansia; mmu-miR-10b-5p was positively correlated with Clostridium. Overall, our study demonstrated significant associations between mmu-miR-1224-5p, mmu-miR-218-5p, mmu-miR-10b-5p, and mmu-miR-488-3p with different bacterial groups.

## 5 Discussion

Gut microbiota composition has been linked to susceptibility to psychiatric disorders, including GAD. However, whether changes in gut microbiota associated with GAD play an etiological role in disease development remains largely unknown. In this study, we transplanted feces from GAD patients into pseudo-germ-free mice and observed that the mice developed anxiety-like behavior accompanied by a significant alteration in flora structure. Interestingly, this alteration mirrored that of the human flora, associated with significant changes in prefrontal cortex miRNAs. The altered composition of the anxiety-associated microbiota was characterized by the absence of Akkermansia and Bifidobacterium bacteria, coupled with enrichment of Allobaculum and Bacteroides. Our findings substantiate that alterations in the gut microbiota of anxious mice lead to changes in prefrontal cortical miRNAs, subsequently influencing related genes and the signaling pathways they participate in. Thus, manipulating the brain through the microbiota emerges as a promising therapeutic avenue for psychiatric disorders such as anxiety.

This investigation confirmed significant changes in the gut microbiota structure of GAD patients through the HAMA score, gut microbiota structural analysis, and other methods. Subsequently, in human-to-mouse FMT, we observed that anxiety-like behavior could be transferred from GAD donors to recipient mice, confirming the pivotal role of gut microbiota in the pathophysiology of GAD. FMT, a treatment involving the transplantation of gut microbiota from a healthy donor into the gastrointestinal tract of another patient to normalize the recipient’s gut microbiota composition (22), has been increasingly explored for its therapeutic potential in various diseases, including inflammatory bowel disease, functional bowel disease, metabolic syndrome, Parkinson’s disease, autism, and more (23). Notably, recent studies have extended FMT applications to mental illnesses such as anxiety and depression. For example, mice receiving FMT from individuals with anxiety and depression exhibited alterations in anxiety and depression-like behavior (24, 25), highlighting the impact of gut microbiota dysbiosis on brain function. As mentioned earlier, gut microbiota can regulate the expression of miRNAs influencing brain function. However, the precise mechanism by which gut microbiota affects miRNA expression profiles in the central nervous system remains elusive and requires further investigation.

We found that the gut microbiota of mice exhibited notable changes following the transplantation of microbiota from patients with GAD, marked by an increase in the abundance of pathogenic bacteria and a decrease in beneficial bacteria. Specifically, the levels of Allobaculum, Bacteroides, and Clostridium genera were elevated in mice that underwent GAD microbiota transplantation (FMT-GAD group), while the abundance of Bifidobacterium, Butyricimonas, and Akkermansia genera decreased. Consistent with our study, compelling evidence suggests that Allobaculum and Bacteroides act as anxiety-inducing microbes in mice (9, 26–28), potentially by reducing brain-derived neurotrophic factor (BDNF) levels (29)in the prefrontal cortex and dopamine expression (30). Conversely, Akkermansia, Bifidobacterium, and Butyricimonas have been associated with anxiolytic effects. Akkermansia, a beneficial gut bacterium, has demonstrated the ability to regulate immune response, reduce intestinal inflammation, and show promise in alleviating mental illnesses. Importantly, direct supplementation of Akkermansia has been linked to increased growth of Bifidobacterium (31) and a reduction in depressive and anxious behavior after chronic stress (32), potentially through the restoration of dopamine and BDNF levels or the modulation of AMPK (33) and PI3K/AKT signaling pathways to inhibit neuroinflammation through CREB (34). Additionally, Bifidobacterium and Butyricimonas can enhance the production of butyrate, an anti-inflammatory substance that impedes the entry of intestinal toxins into the brain. In a study involving patients with irritable bowel syndrome and anxiety treated with Bifidobacterium, an increase in Butyricimonas abundance was observed (35), possibly influencing dopamine receptor expression in areas such as the prefrontal cortex (36). Building upon our study and previous research, we propose that these bacterial genera may engage in intricate crosstalk with AMPK, PI3K/AKT, and dopamine signaling pathways to regulate anxiety-like behavior.

We propose that dysregulation in gut microbiota alters miRNA expression in the prefrontal cortex through the gut-brain axis, potentially contributing to anxiety-like behavior. Our investigation into miRNA regulation involved differential miRNA screening and the construction of miRNA-GO and miRNA-KEGG analysis networks, miRNA-target gene network relationships, and target gene-miRNA-pathway tables. Several biological pathways, such as the AMPK signaling pathway (37), cAMP signaling pathway (38), PI3K-Akt signaling pathway (39), Dopaminergic synapse (40), and Axon guidance (41), commonly associated with anxiety disorders, were found to be regulated by prefrontal cortex miRNA. In the target gene-miRNA-pathway analysis, Creb1 emerged as a key player in all these pathways. Genetic variations in Creb1 have been strongly linked to anxiety disorders in humans (42), and Creb1 mutant mice have been reported to display increased anxiety-like behavior in various behavioral models (43). Our findings indicate Creb1 is modulated by three miRNAs: mmu-miR-10a-5p, mmu-miR-488-3p, and mmu-miR-1224-5p. Notably, miR-10a-5p has been identified as a potential biomarker for acute psychological stress (44, 45) and anxiety by downregulating CREB and BDNF levels (46, 47). miR-488 has been shown to regulate anxiety-related genes (48), and miR-1224-5p expression is significantly reduced under stress, consistent with our results (49, 50). Collectively, our study suggests that mmu-miR-10a-5p, mmu-miR-488-3p, and mmu-miR-1224-5p may modulate anxiety-like behavior through AMPK, cAMP, PI3K-Akt signaling pathways involving Creb1 following fecal transplantation from mice with anxiety disorders. It is highly conceivable that the regulatory effect of mmu-miR-10a-5p, mmu-miR-488-3p, and mmu-miR-1224-5p on anxiety-like behavior induced by fecal transplantation is implemented through Creb1-mediated signaling pathways such as AMPK, cAMP, and PI3K-Akt. This regulatory mechanism may present new targets for the treatment of anxiety disorders, although further experimental validation is necessary to substantiate this hypothesis.

Our study underscores the strong correlation between brain miRNA and gut microbiota, providing evidence for the intimate relationship between the gut-brain axis and anxiety. An inverse relationship observed between mmu-miR-488-3p and Verrucomicrobia and Akkermansia suggests that fecal transplantation in this study may have led to a decrease in the abundance of these bacteria, increasing mmu-miR-488-3p expression. This increase, in turn, may have led to a decrease in Creb1 expression, interfering with the development of AMPK, cAMP, PI3K-Akt signaling pathways, and the Dopaminergic synapse, ultimately regulating anxiety-like behavior.

Few clinical trials have hitherto utilized fecal transplants for the direct treatment of anxiety disorders. Significant challenges in the study of anxiety persist, including the lack of clarity on the exact mechanisms, a scarcity of operable institutions, and low acceptance among relevant populations. The gut microbiota can influence the gut-brain axis and modify brain function through neurological, immunological, and endocrine pathways. We propose that future research should investigate neurotransmitters and metabolites involved in these pathways to elucidate the specific mechanisms of “gut-brain communication” in anxiety. Such investigations would provide a valuable tool for understanding this prevalent psychological disorder with societal implications.

## 6 Conclusion

In summary, FMT in mice induced anxiety-like behavior, marked by a reduction in beneficial bacteria such as Akkermansia and Bifidobacterium, and an increase in pathogenic bacteria like Allobaculum and Bacteroides. This alteration along the gut-brain axis influenced microRNAs, including mmu-miR-488-3p in the prefrontal cortex, ultimately impacting Creb1 and its associated signaling pathways, thereby regulating anxiety-like behavior. These findings emphasize the pivotal role of gut microbiota in GAD. The application of FMT to restore the microbiota of healthy individuals may hold promise in alleviating mental illness and thereby enhancing the quality of life for affected patients.

## Data availability statement

The original contributions presented in the study are included in the article/**Supplementary Material**. Further inquiries can be directed to the corresponding authors.

## Ethics statement

The studies involving humans were approved by Medical Ethics Committee of Beijing University of Chinese Medicine. The studies were conducted in accordance with the local legislation and institutional requirements. The participants provided their written informed consent to participate in this study. The animal study was approved by Experimental Animal Ethics Committee of Beijing University of Chinese Medicine. The study was conducted in accordance with the local legislation and institutional requirements. Written informed consent was obtained from the individual(s) for the publication of any potentially identifiable images or data included in this article.

## Author contributions

SC: Writing – original draft, Writing – review & editing. ML: Writing – original draft. CT: Writing – review & editing. YW: Writing – review & editing. JH: Writing – review & editing. QS: Writing – review & editing. YL: Data curation, Methodology, Supervision, Writing – review & editing. YW: Validation, Writing – review & editing, Supervision. YS: Funding acquisition, Resources, Validation, Writing – review & editing.
