# Supplementary material for: Regulation of miRNA expression in the prefrontal cortex by fecal microbiota transplantation in anxiety-like mice
Source: Front Psychiatry. 2024 Feb 12;15:1323801. doi: 10.3389/fpsyt.2024.1323801 (PMC10894985; doi:10.3389/fpsyt.2024.1323801)
Supplement: Supplementary file 1 [file Table_1.docx]

Supplementary Table 1.Abundance of gut microbiota at the phylum level and different phyla between the groups

| gut microbiota | GAD | Health | Log_2_ FoldChange | Tendency |
| --- | --- | --- | --- | --- |
| (Actinobacteria) | 2483 | 299 | 3.053866872 | ↑ |
| (Gemmatimonadetes) | 15 | 2 | 2.906890596 | ↑ |
| (Deferribacteres) | 95 | 32 | 1.569855608 | ↑ |
| (Proteobacteria) | 1433 | 760 | 0.914967286 | ↑ |
| (Cyanobacteria) | 7 | 5 | 0.485426827 | ↑ |
| (TM7) | 5 | 4 | 0.321928095 | ↑ |
| (Bacteroidetes) | 8077 | 7257 | 0.154446269 | ↑ |
| (Firmicutes) | 25356 | 36276 | -0.516688212 | ↓ |
| (Acidobacteria) | 2 | 3 | -0.584962501 | ↓ |
| (Verrucomicrobia) | 42 | 375 | -3.158429363 | ↓ |

Supplementary Table 2.Abundance of gut microbiota at the genus level and significantly different genera between the groups

| gut microbiota | | GAD | | Health | | Log_2_ FoldChange | | Tendency | |
| --- | --- | --- | --- | --- | --- | --- | --- | --- | --- |
| (Sutterella) | | 473 | | 25 | | 4.241840184 | | ↑ | |
| (Bifidobacterium) | | 2397 | | 154 | | 3.960227653 | | ↑ | |
| (Megamonas) | | 184 | | 20 | | 3.201633861 | | ↑ | |
| Prevotellaceae_Prevotella | | 969 | | 110 | | 3.138993142 | | ↑ | |
| (Lachnospira) | | 2598 | | 416 | | 2.642745997 | | ↑ | |
| (Mucispirillum) | | 95 | | 27 | | 1.814968106 | | ↑ | |
| (Phascolarctobacterium) | | 62 | | 21 | | 1.561878888 | | ↑ | |
| (Bacteroides) | | 3077 | | 1361 | | 1.176857377 | | ↑ | |
| (Coprococcus) | | 275 | | 147 | | 0.903615464 | | ↑ | |
| (Anaerostipes) | | 81 | | 46 | | 0.816288047 | | ↑ | |
| (Allobaculum) | | 28 | | 16 | | 0.807354922 | | ↑ | |
| (Helicobacter) | | 78 | | 48 | | 0.700439718 | | ↑ | |
| (Klebsiella) | | 12 | | 17 | | -0.502500341 | | ↓ | |
| (Clostridium) | | 60 | | 89 | | -0.568842835 | | ↓ | |
| (Dialister) | | 99 | | 154 | | -0.637429921 | | ↓ | |
| (Faecalibacterium) | | 11348 | | 18308 | | -0.690036141 | | ↓ | |
| Ruminococcaceae_Ruminococcus | | 302 | | 496 | | -0.715791571 | | ↓ | |
| AF12 | | 11 | | 19 | | -0.788495895 | | ↓ | |
| (Odoribacter) | | 103 | | 223 | | -1.114399373 | | ↓ | |
| (Streptococcus) | | 11 | | 24 | | -1.125530882 | | ↓ | |
| (Anaerotruncus) | | 34 | | 76 | | -1.160464672 | | ↓ | |
| (Gemmiger) | | 862 | | 1935 | | -1.166573792 | | ↓ | |
| (Exiguobacterium) | | 3 | | 8 | | -1.415037499 | | ↓ | |
| (Parabacteroides) | | 23 | | 77 | | -1.743224585 | | ↓ | |
| (Butyricimonas) | | 4 | | 28 | | -2.807354922 | | ↓ | |
| (Akkermansia) | | 42 | | 310 | | -2.883806982 | | ↓ | |
| (Alistipes) | | 50 | | 488 | | -3.286881148 | | ↓ | |

Note: Only parts of |Log2 FoldChange| > 0.5 are shown.

Supplementary table 3 differential expression of miRNA between the two groups

| miRNA | FoldChange | Pvalue | FMT health/FMT GAD |
| --- | --- | --- | --- |
| mmu-miR-10b-5p | 2.293195532 | 0.00851991 | Up |
| mmu-miR-539-5p | 2.068462126 | 0.028232906 | Up |
| mmu-miR-10a-5p | 1.982540581 | 0.050375879 | Up |
| mmu-miR-300-5p | 1.923686848 | 0.01402221 | Up |
| mmu-miR-146a-5p | 1.862490355 | 0.008380554 | Up |
| mmu-miR-146b-5p | 1.674312816 | 0.023593799 | Up |
| mmu-miR-148a-3p | 1.645267964 | 0.002982898 | Up |
| mmu-miR-467a-5p | 1.62567188 | 0.011426646 | Up |
| mmu-miR-467b-5p | 1.62567188 | 0.011426646 | Up |
| mmu-miR-335-3p | 1.58203147 | 0.019592116 | Up |
| mmu-miR-451a | 1.5605543 | 0.011681076 | Up |
| mmu-miR-30e-3p | 1.553232725 | 0.038405488 | Up |
| mmu-miR-218-5p | 1.498338396 | 0.003304862 | Up |
| mmu-miR-340-3p | 1.48665529 | 0.014432453 | Up |
| mmu-miR-221-5p | 1.457790205 | 0.043418872 | Up |
| mmu-miR-382-3p | 1.456478787 | 0.015979775 | Up |
| mmu-miR-488-3p | 1.450473312 | 0.002971123 | Up |
| mmu-miR-137-3p | 1.437230567 | 0.042658617 | Up |
| mmu-miR-384-5p | 1.419093791 | 0.032785398 | Up |
| mmu-miR-323-5p | 1.437117626 | 0.027081407 | Down |
| mmu-miR-504-5p | 1.463209298 | 0.031159871 | Down |
| mmu-miR-378c | 1.482663952 | 0.006891047 | Down |
| mmu-miR-673-5p | 1.519644001 | 0.027339271 | Down |
| mmu-miR-3470b | 1.631896553 | 0.027236114 | Down |
| mmu-miR-1224-5p | 1.704261841 | 0.006577686 | Down |
| mmu-miR-3102-3p | 1.712992118 | 0.002415015 | Down |
| mmu-miR-690 | 1.74437906 | 0.026490287 | Down |
| mmu-miR-744-5p | 1.756092883 | 0.025712589 | Down |
| mmu-miR-3470a | 1.985240573 | 0.04500042 | Down |
| mmu-miR-6538 | 2.410355205 | 0.009420542 | Down |

Supplementary table 4.miRNA-Gene Degree and corresponding gene

| miRNA | Degree | Gene |
| --- | --- | --- |
| mmu-miR-148a-3p | 105 | Pten、Styx、Scaf11、Usp7、Ago1、Cdk13、Arhgef17  Arpp19、Usp47、Masp1、Mpped1、Sbno1、Fez2 |
| mmu-miR-378c | 69 | Nme6、Psd3、Dcaf12、Chtf8、Nup160、Plekha1  Mpp3、Dyrk1a、Gja8、Dlk1、Fkbp5、Htr1b、Igf1 |
| mmu-miR-1224-5p | 68 | Creb1、Ptprf、Rtl5、Ush1g、Prickle2、N6amt1  Syt14、Med20、Prpf40a、Dhx35、Prc1、Cbx3 |
| mmu-miR-10a-5p | 53 | Flt1、Prrx1、Gnrhr、Wdr26、Slc6a19、E2f3、Ppargc1b  Mapre1、Epha5、Grin2b、Myt1l、Pten |
| mmu-miR-218-5p | 45 | Fam126b、Rps6ka3、Ugt8a、Ssr1、Gpam、Npas2  Pde7a、Camk4、Epha7、Prlr、Sgcd、Ppp1cc |
| mmu-miR-10b-5p | 45 | Med1、Tiam1、Rc3h2、Zmynd11、E2f3、Kctd16  Mapre1、Epha5、Gabrb2、Hoxb3、Elavl3、Irs1 |
| mmu-miR-384-5p | 43 | Lcor、Atp6v0d1、Capza1、Kctd16、Capn5、Ell  Fst、Map3k5、Ppp3ca、Strbp、Zfand5、Celf2 |
| mmu-miR-488-3p | 37 | Grin2b、Mecom、Rad51d、Fign、Rspry1、Mat2a  Cbx5、Gabrb2、Lhx6、Rab7、Rbpj、Calm1 |
| mmu-miR-146a-5p | 36 | Strbp、Meis1、Myo5a、Usp47、Kctd15、Lcor  Foxo3、Kdm2b、Ehf、Itm2b、Syt1、Traf6 |
| mmu-miR-146b-5p | 23 | Kctd15、Zfp532、Strbp、Ehf、Gabrb1、Smad4  Angptl2、Ar、Tef、Ube2d2a、Sfpq、Primpol |
| mmu-miR-137-3p | 14 | Nab1、Pakap、Ptgfrn、Baz1a、Syncrip、Rnf157  Foxp1、Mosmo、Wbp1l、Sez6l2、Tfap2a、Creb5  Pafah1b2、Epha7 |
| mmu-miR-382-3p | 14 | Nrp1、Ccnt1、Map3k19、Foxb1、Fgf16、tga1  Atp13a3、Fam168b、Kcnma1、Camk1d、Sepsecs  1700025G04Rik、Ntrk3、Tfcp2 |
| mmu-miR-744-5p | 13 | Nrgn、Unc5a、Hes3、Rfx1、Mmp24、Tmem167b  Rab14、Camk2n2、Sh3bgrl3、Vgf、Sbf1、Vps37d  Zfp385a |
| mmu-miR-504-5p | 11 | Rnf114、Dcx、Fem1a、Pla2g2f、Crtam、Cutc、Nlrc3  Epb41、Ppargc1b、Ctdspl、Cep170 |
| mmu-miR-539-5p | 7 | Zfp644、Ric3、Hnrnpf、Camsap1、Tanc2、Ctnnd1  Adal |
| mmu-miR-30e-3p | 5 | Pou2f1、Mllt3、Stox2、Galnt7、Spon1 |
| mmu-miR-3470b | 3 | Wee1、Zfyve26、Ddx19b |
| mmu-miR-451a | 3 | Ywhaz、Fign、Zfp644 |
| mmu-miR-221-5p | 1 | Ralgps2 |
| mmu-miR-300-5p | 1 | Wfdc6b |
| mmu-miR-467b-5p | 1 | Ctsb |
| mmu-miR-673-5p | 1 | Vipr2 |

Supplementary table 5 Gene-miRNA Degree and corresponding miRNA

| Target Gene | Degree | FMT health/FMT GAD |
| --- | --- | --- |
| Fign | 4 | mmu-miR-1224-5p↓、mmu-miR-384-5p↑、mmu-miR-488-3p↑、mmu-miR-451a↑ |
| Elavl2 | 4 | mmu-miR-10a-5p↑、mmu-miR-10b-5p↑、mmu-miR-148a-3p↑、mmu-miR-488-3p↑ |
| Creb1 | 3 | mmu-miR-10a-5p↑、mmu-miR-1224-5p↓、mmu-miR-488-3p↑ |
| Epha5 | 3 | mmu-miR-10a-5p↑、mmu-miR-10b-5p↑、mmu-miR-218-5p↑ |
| E2f3 | 3 | mmu-miR-10a-5p↑、mmu-miR-10b-5p↑、mmu-miR-384-5p↑ |
| Strbp | 3 | mmu-miR-146a-5p↑、mmu-miR-146b-5p↑、mmu-miR-384-5p↑ |
| Ppargc1b | 3 | mmu-miR-10a-5p↑、mmu-miR-10b-5p↑、mmu-miR-504-5p↓ |
| Zfp367 | 3 | mmu-miR-10a-5p↑、mmu-miR-10b-5p↑、mmu-miR-146a-5p↑ |
